# Bilateral Optic Neuritis: A Rare Complication of Mumps

**DOI:** 10.7759/cureus.7768

**Published:** 2020-04-21

**Authors:** Beenish Khan, Saad Nasir, Shahina Hanif

**Affiliations:** 1 Ophthalmology, United Medical and Dental College, Creek General Hospital, Karachi, PAK; 2 Internal Medicine, United Medical and Dental College, Creek General Hospital, Karachi, PAK; 3 Pediatrics, United Medical and Dental College, Creek General Hospital, Karachi, PAK

**Keywords:** mumps, optic neuritis, postinfectious optic neuritis, mumps meningoencephalitis

## Abstract

Mumps is a contagious viral illness that classically presents with fever, parotid gland swelling, headache, and vomiting in unimmunized children. The complications of mumps most commonly include orchitis, pancreatitis, encephalitis, and meningitis. Optic neuritis, which refers to the inflammation of the optic nerve, in rare cases, can present after mumps meningoencephalitis and causes pain in the eye, and a decrease in visual acuity. We report and discuss a case of bilateral optic neuritis following mumps meningoencephalitis in a child. The patient was managed with short-term steroid therapy.

## Introduction

Optic neuritis (ON) refers to a demyelinating inflammatory condition involving the optic nerve. It typically presents with sub-acute unilateral vision loss, associated with painful eye movements. Optic neuritis in the majority of cases is idiopathic, while it is commonly associated with conditions that include multiple sclerosis, neuromyelitis optica (NMO) and less commonly with autoimmune diseases, infectious diseases and following vaccination [1]. Although rare, children most frequently develop bilateral ON, as compared to unilateral ON in adults, and often follows viral infections such as *mumps, measles, rubella,* and *influenza* [2-3]. The prevalence of optic neuritis is twice as high in England as in the United States, while it is significantly less prevalent in Eastern countries [4]. Here, we present a case of bilateral optic neuritis in a 10-year-old patient secondary to mumps meningoencephalitis.

## Case presentation

A 10-year-old girl brought by her mother to the emergency department presented with the complaint of sudden onset blurring of vision. She had a history of hospital admission two days before this episode and was admitted for the management of viral meningitis. The patient at the time presented with a history of fever and bilateral swelling of the parotid region for a week with associated headache and vomiting. Pertinent examination findings included bilateral parotid gland swelling accompanied by cervical lymphadenopathy. On central nervous system examination, signs of meningeal irritation were positive. Cerebrospinal fluid (CSF) analysis was subsequently done, and it showed increased white blood cell count, normal glucose, and slightly increased protein levels, which were consistent with viral meningitis. Based on CSF analysis and clinical presentation, the diagnosis of mumps meningoencephalitis was confirmed. She received supportive therapy during the hospital stay and was discharged 10 days later.

**Figure 1:**
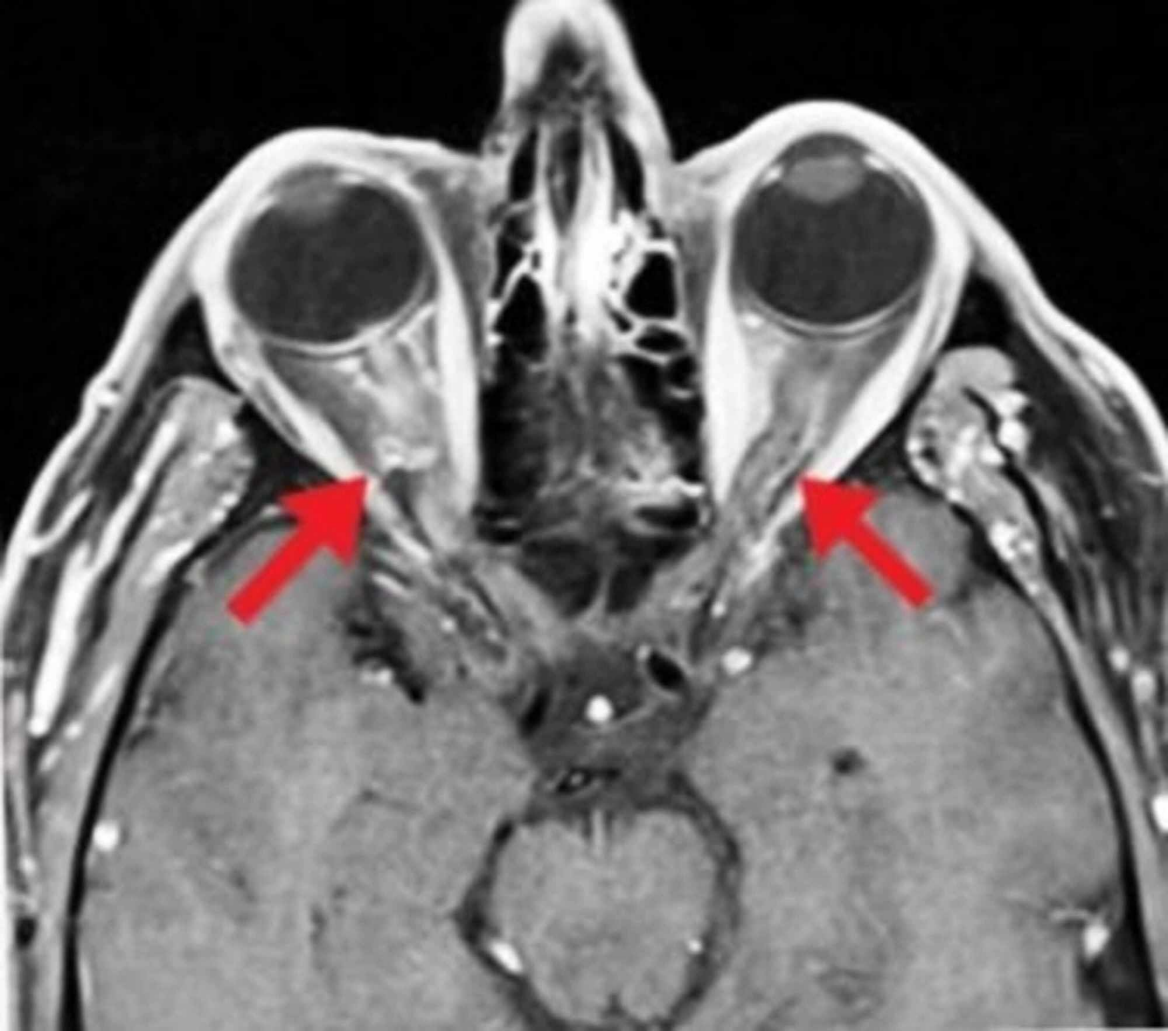
Magnetic resonance imaging of the brain showing bilateral optic nerve thickening

The general physical examination now revealed an irritable child with intact orientation. Her vitals were normal. Ophthalmology consult was taken, which showed visual acuity of 6/6 in the right eye and 6/60 in the left eye. There was a mild relative afferent pupillary defect in the left eye, whereas the color vision of both eyes was also defective. Anterior segment examination was within the normal range. Fundoscopy revealed bilateral optic disc edema with the blurring of the optic disc margins accompanied by few splinter hemorrhages around the discs. These findings were more prominent on the left side as compared to the right side. The central nervous system examination was unremarkable. Magnetic resonance imaging (MRI) of the brain without contrast revealed thickening of both optic nerves (Figure 1). The diagnosis of bilateral optic neuritis secondary to mumps meningoencephalitis was confirmed.

The patient was immediately started on intravenous methylprednisolone 750 mg/day for three days, followed by oral prednisolone 5 mg at a dose of 1 mg/kg. Her follow-up a week later showed gross vision improvement in the left eye from 6/60 to 6/6, although clinically, there were still signs of optic disc edema; in resolving stage. After 11 days of treatment, clinical signs of optic neuritis disappeared, with no residual disc pallor. Steroids were tapered gradually over the next 20 days. The patient has since recovered with no clinical signs of optic neuritis in both eyes and has resumed her routine daily life.

## Discussion

Mumps is an acute transmissible viral illness caused by a single-stranded RNA paramyxovirus. According to the World Health Organization (WHO), the annual incidence of mumps worldwide (excluding the countries with more aggressive vaccination) is 100 to 1000 cases per hundred thousand population [5]. It manifests as unilateral or bilateral swelling of the parotid glands after a viral prodrome. The most common clinical features include fever, bilateral parotid gland swelling, headache, nausea, vomiting, and sometimes seizures. The neurological complications linked with mumps include sudden-onset sensorineural hearing loss, aseptic meningitis, which is usually benign and encephalitis, which is associated with grave consequences, including death [6]. Ocular involvement may lead to follicular conjunctivitis, episcleritis, dacryoadenitis, keratitis, scleritis, anterior uveitis, choroiditis, and paralysis of extraocular muscles (abducens nerve palsy), although rare, there can also be bilateral optic neuritis after an encounter with mumps virus [7]. Mumps is preventable with the administration of themeasles-mumps-rubella (MMR) vaccine, with effectiveness after a single dose ranging from 49-92% (median: 78%), and to 66-95% (median:88%), after administration of the second dose [6].

Optic neuritis (ON) is defined as the inflammation of the optic nerve presenting as a painful eye and decreased visual acuity. Papillitis, neuro-retinitis, and retrobulbar neuritis are the three forms of ON [8]. Banwell et al. estimated the incidence of pediatric ON in Canada to be 0.2 cases per million, but there is no other study to compare the incidence of this condition with other countries [9]. It can occur following infection with various pathogens including viruses (including *influenza, mumps, *and *measles*), bacteria (including* Bartonella henselae, tuberculosis, syphilis,* and *Borrelia burgdorferi*), fungi (including *cryptococci, candida, Histoplasma, aspergillus, *and* mucormycosis*) and parasites (including *Toxoplasma gondii, Toxocara canis, Onchocerca volvulus, malaria, *and *Echinococcus*), and is considered atypical [3]. A review done by Perez-Cambrodi et al. proposed that bilateral optic neuritis in children is associated with a good prognosis as compared to adults, and suggested the use of short-term corticosteroids for the management of pediatric patients with poor bilateral visual acuity [10].

To the best of our knowledge, this is the first report of bilateral optic neuritis secondary to mumps meningoencephalitis in Pakistan. There have been similar pediatric ON reports from other regions, including India (seven-year-old girl), Japan (five-year-old boy), and the United States (seven-year-old girl) [11-13].

A retrospective study done by Brady et al. to determine the clinical outcomes features and outcomes of pediatric ON included 25 patients (mean age of presentation 9.4 years), out of which, 14 patients (54%) had bilateral ON and showed evidence of marked improvement on steroid therapy [14]. Gnananayagam et al. presented two cases (4.5-year-old girl and a 10-year-old boy) of optic neuritis (retrobulbar) following mumps infection, which resolved with steroid therapy [8].

In this article, we highlight bilateral optic neuritis as a rare complication of mumps. There is limited data to suggest the role of steroids for the treatment of optic neuritis in the pediatric population, and through this report, we further strengthen the literature by reviewing the findings of previous studies and providing substantial evidence of the associated benefit with short-term steroid therapy in rapid regression of this condition.

## Conclusions

Postinfectious optic neuritis in the pediatric population is a rare complication, and in this report, we describe a case of optic neuritis secondary to mumps meningoencephalitis. Physicians should be aware of this complication as early management with steroids can hasten the disease progression, and the rapid return of visual acuity ensures with no residual vision impairment.
